# Positive and Negative Regulation of Prostate Stem Cell Antigen Expression by Yin Yang 1 in Prostate Epithelial Cell Lines

**DOI:** 10.1371/journal.pone.0035570

**Published:** 2012-04-19

**Authors:** Shuai Tang, Meenu Mishra, Donna P. Frazier, Miranda L. Moore, Kazushi Inoue, Rajendar Deora, Guangchao Sui, Purnima Dubey

**Affiliations:** 1 Molecular Pathology Graduate Program, Wake Forest School of Medicine, Winston-Salem, North Carolina, United States of America; 2 Department of Pathology-Tumor Biology, Wake Forest School of Medicine, Winston-Salem, North Carolina, United States of America; 3 Department of Cancer Biology, Wake Forest School of Medicine, Winston-Salem, North Carolina, United States of America; 4 Comprehensive Cancer Center, Wake Forest School of Medicine, Winston-Salem, North Carolina, United States of America; 5 Department of Microbiology and Immunology, Wake Forest School of Medicine, Winston-Salem, North Carolina, United States of America; University of Medicine and Dentistry of New Jersey, United States of America

## Abstract

Prostate cancer is influenced by epigenetic modification of genes involved in cancer development and progression. Increased expression of Prostate Stem Cell Antigen (PSCA) is correlated with development of malignant human prostate cancer, while studies in mouse models suggest that decreased PSCA levels promote prostate cancer metastasis. These studies suggest that PSCA has context-dependent functions, and could be differentially regulated during tumor progression. In the present study, we identified the multi-functional transcription factor Yin Yang 1 (YY1) as a modulator of PSCA expression in prostate epithelial cell lines. Increased YY1 levels are observed in prostatic intraepithelial neoplasia (PIN) and advanced disease. We show that androgen-mediated up-regulation of PSCA in prostate epithelial cell lines is dependent on YY1. We identified two direct YY1 binding sites within the PSCA promoter, and showed that the upstream site inhibited, while the downstream site, proximal to the androgen-responsive element, stimulated PSCA promoter activity. Thus, changes in PSCA expression levels in prostate cancer may at least partly be affected by cellular levels of YY1. Our results also suggest multiple roles for YY1 in prostate cancer which may contribute to disease progression by modulation of genes such as PSCA.

## Introduction

Prostate cancer is a heterogeneous disease arising from genetic events such as *Pten* deletion which result in tumor initiation [1], [2], [3]. Epigenetic gene regulation may augment tumor initiation in conjunction with the oncogenic signal and is known to modulate tumor progression [4]. Thus it is critical to understand transcriptional and translational control mechanisms which influence tumor progression, as these pathways may provide novel therapeutic opportunities for advanced disease.

Prostate Stem Cell Antigen (PSCA) is a GPI-anchored cell surface protein [5] and is a marker of the transiently amplifying cell population within prostate epithelium [6]. PSCA is also expressed in epithelial cells of various organs including the kidney, bladder, stomach and pancreas [7]. PSCA over-expression is reported in a subset of prostate cancers at all stages from PIN to metastatic disease [8]. Although this protein has been considered as a target for therapy [8], [9] and imaging [10] of prostate cancer, its function is still unknown. Studies of human prostate cancers suggest that expression of PSCA in PIN is a predictor of later development of invasive adenocarcinoma [11]. In addition our studies in a murine prostate cancer model showed that loss of PSCA promotes tumor metastasis [12]. Together, these data suggest that changes in PSCA expression levels may alter tumor development and progression.

PSCA is an androgen-responsive gene and expression in the prostate is modulated in response to systemic changes in androgen, through interaction of androgen receptor (AR) with an androgen-responsive element (ARE) [13]. However, other control mechanisms must be involved, since PSCA is expressed in castration-resistant prostate cancer [5], and in androgen-insensitive organs such as the kidney, stomach, pancreas and bladder [7].

The transcription factor Yin Yang 1 (YY1) [14] is expressed in normal tissues and is up-regulated in various cancers including prostate cancer, with positive and negative regulatory effects on gene expression [15], [16]. Elevated YY1 expression is correlated with development of PIN and advanced prostate cancer [15], [17], [18]. Although the function of YY1 in prostate cancer is not fully known, it was reported recently that YY1 forms a complex with AR, which together binds to the ARE within the PSA promoter, stimulating gene expression [19]. Thus, YY1 has a role in epigenetic regulation of prostate cancer genes.

In this study, we demonstrated that androgen-mediated stimulation of PSCA expression requires YY1. In addition, we identified two direct YY1 binding sites within the upstream region of the PSCA gene. Deletion analysis showed that the upstream site represses PSCA promoter activity, while the downstream site stimulates promoter activity. Furthermore, knockdown of YY1 in prostate cancer cell lines increased endogenous PSCA message, suggesting that changes in PSCA message levels during prostate cancer progression may be at least partially regulated by YY1.

## Materials and Methods

### Cell lines

Pten P8 and Pten CaP8 cells were provided by Dr. Hong Wu (UCLA) and maintained as described [20]. LNCaP cells [21] provided by Dr. Owen Witte (UCLA) and PC-3 cells (ATCC, [22]) were maintained in RPMI with 10% fetal bovine serum. HEK293T cells [23] were a gift of Dr. Owen Witte.

### PSCA promoter constructs

The murine PSCA genomic clone was a gift of Dr. Owen Witte. The −4.5 kb EcoRI promoter fragment was isolated by restriction digest from pBluescript and cloned into the EcoR1 site of pGLuc-Basic (Promega). To generate site-specific mutations of the YY1 binding sites, a 2 kb BamHI fragment of the promoter isolated from pGLuc-4.5.kb was sub-cloned into pBluescript. Mutations were performed essentially as described in the Stratagene Quik-change PCR kit using the following primers: mPSCA Site 1 primer: 5′-GGCTTTAACTTCCAGAACCCA***ACGCGT***GGACCATAGTGGGAGAGG-3′ and mPSCA Site 2 primer 5′-CCCTGGGACAGCTGG***GTCGAC***CTCTGCCCGTGATTC-3′. The mutated nucleotides are underlined.

A MluII restriction site was introduced in mPSCA Site 1 and a Sal I site was introduced in mPSCA Site 2 (restriction sites bold and italicized) to facilitate identification of colonies containing the mutations, and was confirmed by sequencing of the fragment. The BamHI fragments containing one or both of the mutations were replaced into the pGLuc-4.5 kb promoter construct.

### Production and purification of recombinant YY1 protein

The HA-YY1 cDNA was PCR amplified from pcDNA3.1-HA-YY1using *Pfu Ultra* DNA polymerase (Stratagene, Agilent Technologies) with sense primer 5′-AGTCCGGAATTC **GCCACC** *ATG*TGCTACCCATACGATGTTC-3′ and antisense primer 5′-AGTCCGGAATTC *TCA*CGGCTGGTTGTTTTTGGCCTTAGCA-3′ (EcoRI sites are underlined; Kozak's consensus sequence is bolded; ATG and stop codon are italicized), digested with EcoRI and ligated into the unique EcoRI site of the pVL1392 baculovirus transfer vector (BD Biosciences). HA-YY1 baculovirus stock was prepared following manufacturer's instructions by co-transfecting *Spodoptera frugiperda* Sf9 cells with 4 µg of pVL1392-HA-YY1 and 0.5 µg of baculovirus gold DNA (BD Biosciences) per 2×10^6^ cells/60 mm. Sf9 cells were maintained in Grace's medium containing 10% FBS, Yeastolate, lactalbumin hydrolysate, and gentamicin (Invitrogen). Low titer stock was harvested and 40 µL was infected into 1×10^7^ Sf9 cells for amplification, and high titer HA-YY1 baculovirus stock was harvested and stored at 4°C until use. For HA-YY1 protein expression, 500 µL of the HA-YY1 baculovirus stock was used to infect 1×10^7^ Sf9 cells/10 mL. Forty-eight hours later, the infected cells were harvested in EBC buffer plus protease inhibitors [24]. HA-YY1 was isolated from the Sf9 lysate using EZview Red Anti-HA Affinity Gel (Sigma) according to manufacturer's instructions and analyzed by SDS-PAGE. Protein concentration was determined using protein standards (BioRad). HA-YY1 was verified by Western blotting with monoclonal YY1 antibody (H-10, Santa Cruz Biotechnology).

### Electrophoretic Mobility Shift Assay (EMSA)

EMSA was performed as described previously [25], [26]. A 500-bp fragment encompassing each binding site was generated by PCR and cloned into pBluescript using the KpnI and EcoRI enzymes in the primer sequences. The plasmids were first digested with Kpn I, end-labeled with γ-^32^P-dATP using T4 polynucleotide kinase (New England Biolabs), and then digested with EcoRI. Excess unincorporated radioactivity was removed by purification through a G-50 quick-spin column (GE Healthcare). Reactions containing the radiolabeled fragment, purified HA-YY1 protein and 1× binding buffer [10 mM Tris-HCl (pH 7.8), 2 mM MgCl2, 50 mM NaCl, 1 mM dithiothreitol, 100 ng of bovine serum albumin, 10% glycerol] in 20 µl volume, were incubated at 37°C for 15 min to allow DNA-protein binding. The samples were electrophoresed on a 5% polyacrylamide gel and visualized by autoradiography.

### Chromatin immunoprecipitation

Chromatin immunoprecipitation (ChIP) assays were carried out as described previously [27]. Precipitated DNA was analyzed by PCR using primers specific for the human and murine upstream and downstream YY1 binding sites. Human PSCA downstream site (hPSCA1) primers: FWD-5′-GTGGTGGCCTTTCGTTAGC-3′ and REV-5′-GGCCCTGTCAAATGCCACTC; human upstream site (hPSCA2) primers: FWD-5′-GGACGGGATCACTTGGTCTC-3′ and REV-5′-GGGCTGAGAGGCTGATGTGA-3′. Murine PSCA downstream site (mPSCA1) primers: FWD-5′CGGGGTACCGCACTGTGAGCCAAAACC-3′ and REV-5′-CCCAAGCTTGGCGTCATGTGCCAAGC-3′; murine upstream site (mPSCA2) primers: FWD-5′-GCCTGTGGAGCTTCAGCCCA-3′ and REV-5′-CAGACCTCTACCTCTTCT-3′. Beta actin primers used as negative control were: FWD-5′-CACAGGCATTGTGATGGACT-3′ and REV-5′-CTCACAGGATGCAGAAG-3′.

PCR products were analyzed on a 1% agarose gel and visualized by ethidium bromide.

### Lentiviral infection

LNCaP or PC3 cells were plated in 6-well plates (Corning) at a density of 1×10^5^ cells/well, 12–18 hours prior to infection in their respective culture medium. The next day, concentrated lentivirus was added to the well at a 1∶100 dilution with 8 µg/ml polybrene (Sigma). The virus was removed after 8–15 hours of incubation and fresh medium was added to the wells. Cells were trypsinized and harvested for analysis 48–60 hours following infection.

### R1881 stimulation

For stimulation with synthetic androgen, cells were cultured for 24 hr in Pten medium prepared using phenol-red free DMEM with 10% charcoal-stripped serum. Androgen analog R1881 (10 nM final in DMSO) or DMSO alone was added for an additional 24 hr of culture [19].

### Transient transfection

HEK293T cells were transiently transfected in triplicate with 3 µg of DNA (2.5 µg pGLuc2 plasmids containing the intact promoter or the site-specific mutations, with 0.5 µg dsRed-containing plasmid for transfection control) using GeneJuice (Novagen) and the manufacturer's protocol. Expression of dsRed and luciferase activity was determined 72 hr post-transfection.

### Quantitative real-time PCR analysis

RNA was extracted from prostate cancer cell lines using the Qiagen RNEasy isolation kit and manufacturer's instructions. Human and murine PSCA message levels were determined using TaqMan Gene Expression assay kits (Applied Biosystems) and following manufacturer's instructions. Analysis was performed on an ABI 7700 real-time PCR machine, using the relative quantitation method. Normalization was performed using human GAPDH and murine beta actin, also using TaqMan Gene Expression assay kits.

### Luciferase assays

Infected cells were lysed in Passive Lysis Buffer (Promega) and Gaussia luciferase activity was assessed using 2 µg/ml coelenterazine in DMEM as a substrate, and read in a luminometer. Luciferase activity was normalized to the percentage of dsRed-expressing cells in each well.

### Flow cytometry

Cell surface PSCA protein levels were quantified by staining cell lines with rabbit polyclonal anti-PSCA antibody (0.2 µg/ml, Santa Cruz Biotechnology) followed by goat anti-rabbit-APC (1∶1000) and analyzed on a BD Facscalibur instrument using Cellquest Pro® software. Expression of dsRed in live cells was analyzed on the FACSCalibur.

## Results

### Recombinant YY1 directly binds to predicted binding elements in the human and murine PSCA promoters

PSCA responses to systemic androgen are mediated through an androgen-responsive element (ARE) in the human and murine promoter regions. However, regulatory mechanisms which modulate PSCA expression in the prostate in the absence of androgen, or in other epithelial tissues, are undefined. In order to identify other putative control mechanisms, we scanned the human and murine PSCA sequences using the TFSearch algorithm [28] to determine whether other transcription factors with a role in epithelial cancer development and progression may also interact with the PSCA promoter. A previous study showed that expression in a transgenic mouse of a Green Fluorescent Protein (GFP) reporter gene behind −9 kb PSCA upstream region replicated the expression pattern of endogenous PSCA [29]. Subsequent work showed that the shorter −6 kb upstream region [13] maintained promoter activity and specificity and contained the ARE. Therefore, we limited our analysis to the −6 kb regions of the human and murine PSCA promoters.

**Figure 1:**
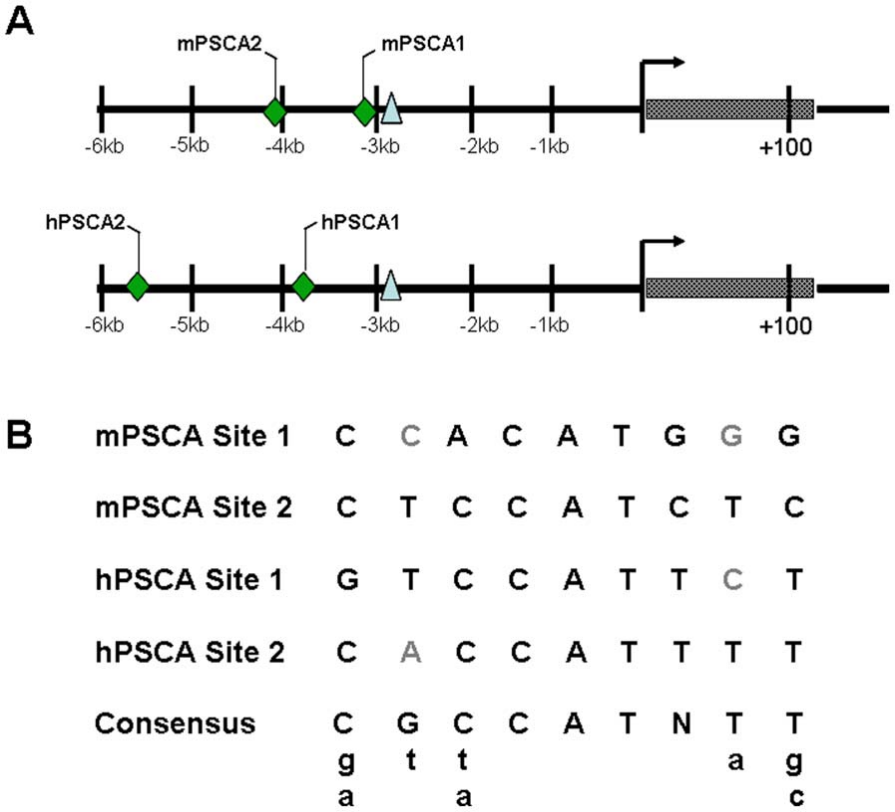
Schematic diagram of predicted YY1 binding sites in the human and murine PSCA promoter. **A.** Two YY1 binding sites (shown as diamonds) predicted by the TFSearch algorithm are located upstream of an ARE (triangles) in both the human and murine promoter; **B.** Nucleotide sequence alignment of these sites to consensus YY1 binding sites [30] shows significant homology.

We identified two predicted YY1 binding sites within both of these sequences. Figure 1A shows the relative location of the binding sites compared to the previously identified ARE within these promoters [13], and in relation to the transcription initiation site previously identified for the hPSCA promoter. The nucleotide sequence of the sites and homology to the consensus YY1 site [30] is shown in Figure 1B. The downstream site, mPSCA1 is adjacent to the ARE. No putative ARE was identified adjacent to the upstream site, mPSCA2.

**Figure 2:**
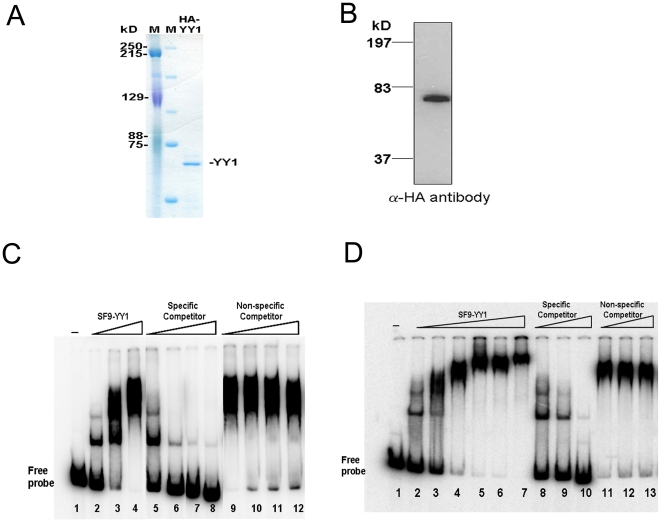
Recombinant YY1 protein binds specifically to murine PSCA promoter fragments. **A.** Coomassie stained polyacrylamide gel shows purity of HA-tagged YY1 protein produced in Sf9 insect cells. **B.** Western blot using anti-HA antibody confirms size and integrity of recombinant YY1. **C.** EMSA analysis using a 500 bp fragment including the downstream YY1 binding site (mPSCA1) in the murine promoter shows specific binding of YY1 to this fragment. Specific competitor is unlabeled promoter fragment and non-specific competitor is poly dIdC. **D.** EMSA analysis using a 500 bp fragment including the upstream YY1 binding site (mPSCA2) in the murine promoter shows specific binding of YY1 to this fragment. Specific competitor is unlabeled promoter fragment and non-specific competitor is poly dIdC.

To determine whether both of the putative YY1 binding sites in the murine PSCA promoter bound purified YY1 *in vitro*, we conducted EMSA analysis. We used recombinant human HA-tagged YY1 for our studies, since human and murine YY1 proteins share 97% similarity, with 100% identity in their zinc finger DNA-binding domains. Recombinant HA-YY1 was produced in SF9 insect cells, purified using the HA tag (Figure 2A) and verified by Western blot (Figure 2B). Binding of the recombinant protein to the downstream predicted site (mPSCA1) (Figure 2C) and the upstream site (mPSCA2) (Figure 2D) was observed and was competed by non-radioactive specific DNA but not by poly dI-dC as non-specific competitor. At higher concentrations of SF9-YY1 protein, there was no change in the mobility of the DNA-protein complex for mPSCA2. Therefore fewer lanes are shown in Figure 2C than in Figure 2D.

### Chromatin immunoprecipitation assay using anti-YY1 antibody detects human and murine PSCA upstream sequences from prostate cancer cell lines

**Figure 3:**
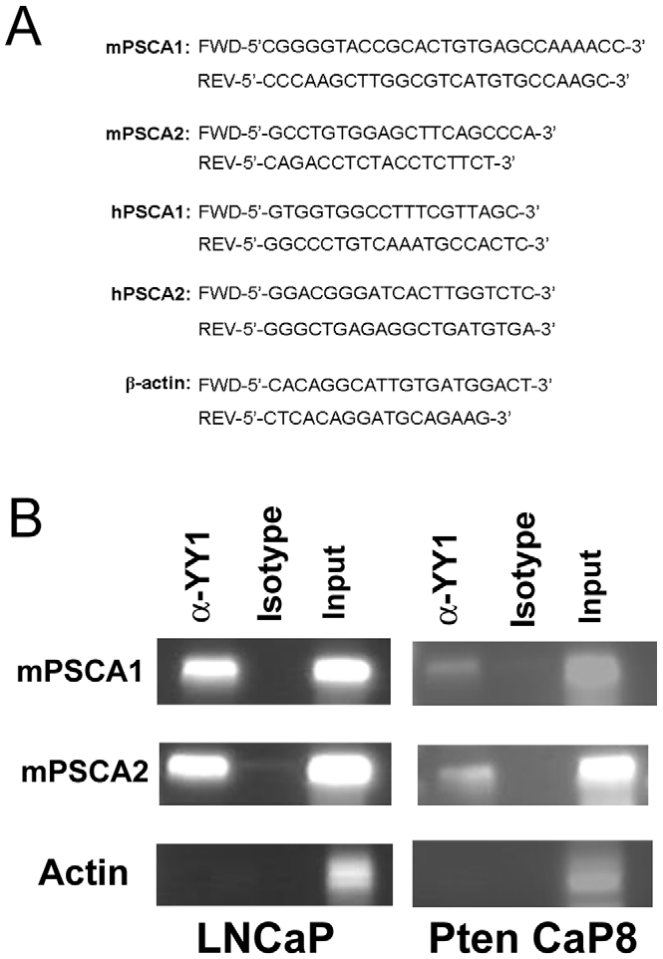
Chromatin immunoprecipitation analysis detects binding of YY1 to the human and murine PSCA promoter. **A.** PCR primers flanking the predicted upstream and downstream YY1 binding sites for murine and human predicted YY1 binding sites and the control β-actin are shown. **B.** The cross-linked YY1-DNA complex was immunoprecipitated with a murine anti-YY1 monoclonal antibody or an isotype control antibody. The YY1 binding DNA fragment was amplified from both samples using the primers shown in *A* and visualized by agarose gel electrophoresis. Input samples were loaded as a control.

To determine interaction of YY1 with the PSCA promoter *in vivo*, we conducted chromatin immuno-precipitation analysis (ChIP) using human and murine prostate cancer cell lines. LNCaP cells are a human androgen-dependent prostate cancer cell line [21]. The Pten CaP8 cell line was derived from the prostate tumor which developed in a prostate-specific *Pten* knockout mouse [20]. Immunoprecipitation was conducted using a murine anti-YY1 monoclonal antibody and genomic DNA was analyzed by PCR. The upstream and downstream YY1 binding sites were specifically immunoprecipitated in both LNCaP and Pten CaP8 cell lines, demonstrating interaction of endogenous YY1 with the PSCA promoter (Figure 3). Thus, interaction of YY1 with the PSCA promoter occurs *in vivo*, and has the potential to modulate PSCA expression in human and murine prostate cancer cell lines.

### Mutation of the direct YY1 binding sites modulates mPSCA promoter activity

**Figure 4:**
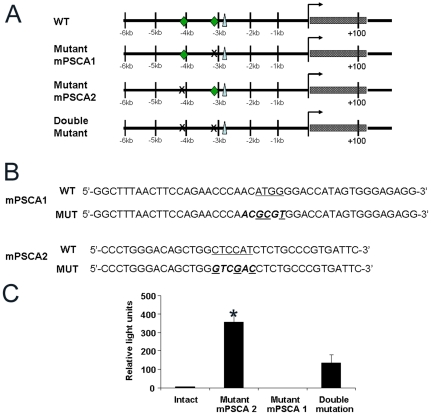
Mutation of YY1 binding sites mPSCA1 and mPSCA2 has opposing effects on PSCA promoter activity. **A.** Schematic representation of constructs with mutations of the YY1 binding sites in the mPSCA promoter (♦, YY1 binding site, ▴, ARE, X, site of mutation). **B.** The primer sequences used to generate the mutations are shown. A restriction site (bold and italicized) was introduced to identify the mutation and is shown in the lower line (labeled MUT) for each binding site. The mutated nucleotides are underlined. **C.** HEK293T cells were transiently transfected with the intact or mutant promoter constructs along with dsRed expressing vector as transfection control. Cells were harvested 72 hr later and analyzed by flow cytometry for dsRed expression. Gaussia luciferase activity in cell supernatant was measured and normalized to the percentage of dsRed positive cells (data not shown). Each infection was done in triplicate. One representative experiment is shown and the experiment was repeated twice. *, p<0.005 compared with intact construct; p<0.03 compared with upstream and downstream mutations.

YY1 binding to promoters can either inhibit or stimulate transcription [31], [32]. To determine whether YY1 binding positively or negatively regulated PSCA promoter activity, we generated reporter constructs where 4.5 kb of the mPSCA promoter controlled expression of the Gaussia luciferase reporter. Site-specific mutations of mPSCA1 and mPSCA2 were introduced alone or together into the −4.5 kb promoter. HEK293T cells were transiently transfected with the reporter constructs along with a dsRed-expressing vector to determine transfection efficiency. Transfected cells were analyzed 72 hr later. Mutation of three nucleotides in mPSCA2 increased luciferase activity several hundred-fold compared to the intact construct (Figure 4). In contrast, mutation of mPSCA1 by changing 3 nucleotides repressed promoter function (Figure 4). Mutation of both binding sites generated intermediate luciferase activity, showing opposing functions for the two binding sites. Thus, YY1 may exert dual control on PSCA promoter expression.

### YY1 is required for R1881-mediated stimulation of PSCA expression

The binding of AR to ARE sequences within promoters can either stimulate or inhibit gene transcription and is dependent on the cofactors recruited to the site. It was recently reported by one of us [19] that AR stimulation of PSA transcription requires YY1, and is mediated by an AR-YY1 protein complex which binds to the ARE. mPSCA1 is separated from the previously defined AREs within the PSCA promoter by approximately 200 nucleotides. To determine whether AR-mediated stimulation of PSCA expression also required YY1, we investigated the effect of YY1 knockdown on endogenous PSCA protein and message levels.

**Figure 5:**
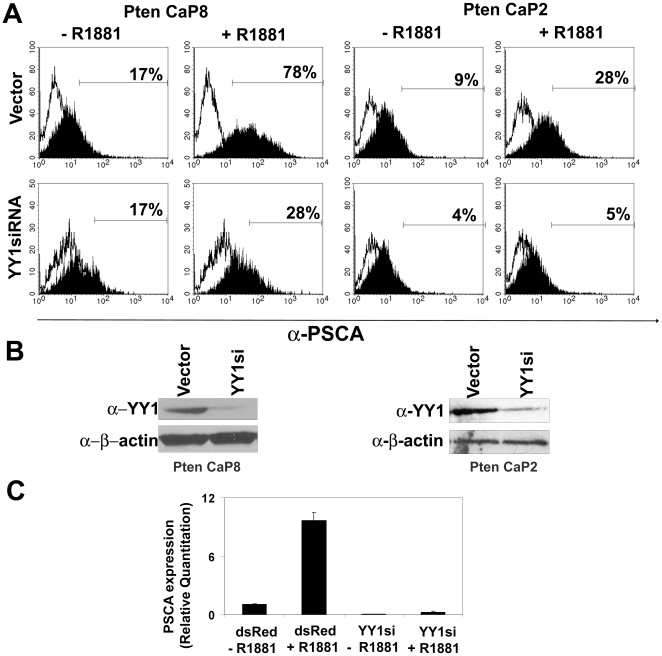
Knockdown of YY1 reduces androgen-mediated up-regulation of PSCA on the cell surface. **A.** Pten CaP8 and Pten CaP2 cells were incubated in hormone-depleted medium for 48 hours and then treated with 10 nM R1881 or vehicle (upper panels). Pten CaP2 and Pten CaP8 cells were infected with a lentivirus expressing YY1 specific siRNA or dsRed expression virus as control. Cells were then androgen deprived and treated with 10 nM R1881 for 48 hours (lower panels). Cell surface PSCA expression was determined by FACS using a polyclonal rabbit anti PSCA antibody. **B.** Expression of YY1 in Pten CaP8 and Pten CaP2 cells infected with YY1 siRNA expressing or control lentivirus was determined by Western blot. The blot was re-probed with anti-β-actin as a loading control. **C.** Quantitative real-time PCR analysis for PSCA expression was conducted on RNA extracted from Pten CaP2 cells in A.

Pten CaP8 and Pten CaP2 cells are cell lines derived from the tumors of prostate-specific *Pten* knockout mice [20]. Pten CaP8 and Pten CaP2 cells were cultured in androgen-depleted conditions for 48 hours. The androgen analog R1881 or vehicle was added to the cells and cell surface PSCA expression was determined 48 hours later by flow cytometry. PSCA expression was increased following R1881 stimulation of Pten CaP8 and Pten CaP2 cells (Figure 5A, upper panels). To determine whether knockdown of endogenous YY1 influenced stimulation of PSCA protein levels by R1881, the cells were infected with a lentivirus expressing YY1siRNA [33] and GFP or with GFP-expressing virus alone followed by androgen deprivation and stimulation of the infected cells. Infection with the YY1 knockdown construct resulted in a 50% reduction in cell-surface PSCA levels following R1881 stimulation compared with vector infected Pten CaP8 cells (Figure 5A, lower panel), while YY1 knockdown abrogated the increase in cell-surface PSCA levels in Pten CaP2 cells. Knockdown of YY1 was achieved with greater than 90% efficiency in both cells (Figure 5B). Thus, YY1 expression is necessary for androgen-mediated stimulation of PSCA expression. Both Pten CaP2 and Pten CaP8 cells die 96 hours following YY1siRNA infection (data not shown) suggesting that YY1 knockdown adversely affects viability of these cells.

To determine whether the change in PSCA protein was a result of transcriptional modulation, Pten CaP2 cells infected with the YY1siRNA construct, or dsRed vector alone, were evaluated for changes in endogenous PSCA message levels. Real-time quantitative PCR analysis showed that PSCA message levels were reduced by knockdown of YY1 (Figure 5C), and a slight, but not statistically significant increase (p = 0.09) was observed following R1881 stimulation of the YY1siRNA infected cells. This increase in PSCA message could be the result of better cell viability in hormone-replete medium, or stimulation of expression from the residual YY1 present in the cells.

### YY1 inhibits PSCA expression in androgen-independent prostate cancer cells

**Figure 6:**
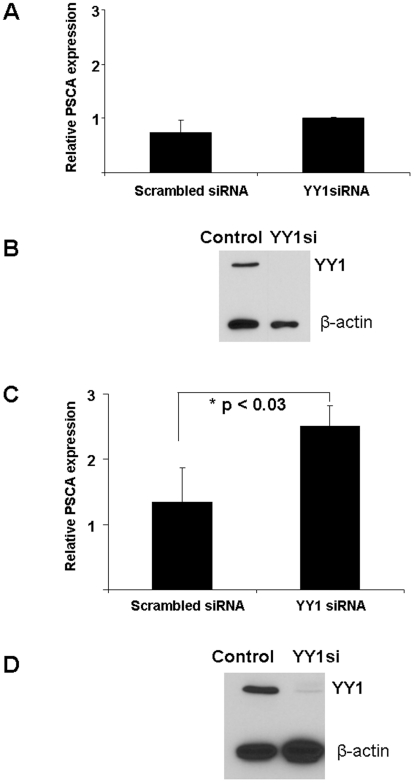
YY1 knockdown in an androgen-independent prostate cancer cell line upregulates endogenous PSCA message levels. **A.** LNCaP cells (2×10^5^/well) were infected in triplicate with YY1siRNA or scrambled control, and evaluated 72 hr later. PSCA message was determined by quantitative real-time PCR analysis. **B.** The amount of endogenous YY1 protein in YY1siRNA- or scramble control-infected cells was determined by Western blot. Representative analysis of one sample of triplicates is shown. **C.** PC-3 cells (2×10^5^/well) were infected in triplicate with YY1siRNA or scrambled control, and evaluated 72 hr later. PSCA message was determined by quantitative real-time PCR analysis. One independent repeat of the experiment was conducted with similar results. * = p<0.03. **D.** Endogenous YY1 protein in YY1siRNA- or scramble control-infected cells was determined by Western blot. Representative analysis of one sample of triplicates is shown.

To determine whether YY1 also modulates human PSCA expression, we determined the effect of YY1 knockdown in an androgen-dependent human prostate cancer cell line, LNCaP, and an androgen-independent human prostate cancer cell line, PC-3. LNCaP cells were infected with YY1siRNA lenvirus or dsRed control virus, and RNA was purified 72 hr later. There was no significant change in human PSCA message levels in LNCaP cells (Figure 6A), despite nearly 100% infection of the cells (not shown) and efficient knock down of YY1 protein (Figure 6B). These results suggest that in a cell line with functioning androgen receptor, the positive and negative effects of YY1 are cancelled out by knockdown of YY1, resulting in no net change in PSCA expression levels.

To determine whether YY1 regulates PSCA expression without the contribution of AR, the effect of YY1 knockdown was evaluated in PC-3 cells, which contain mutant AR. PC-3 cells were infected with the YY1siRNA lentivirus or dsRed control virus, and RNA was purified 72 hr later. Quantitative RT-PCR analysis showed that infection with YY1siRNA significantly increased endogenous PSCA expression in PC-3 cells (Figure 6C). Nearly 100% of the cells were infected (data not shown) resulting in effective knockdown of YY1 protein expression (Figure 6D). These data suggest that YY1 represses PSCA expression in androgen-independent prostate cancer cells.

## Discussion

Levels of PSCA protein and message vary in prostate cancer and may be affected by different modifiers during tumor progression. Previous work showed that PSCA expression is reduced after castration [29], [34], and increased when androgen is provided exogenously, likely due to binding of androgen to an ARE within the PSCA promoter [13]. In this study, we demonstrated that the multifunctional transcriptional factor YY1 binds directly to the PSCA promoter and regulates PSCA expression in androgen-dependent and independent cell lines. The increase in cell surface PSCA protein levels following stimulation with a synthetic androgen analog was regulated by YY1. We observed a partial reduction in cell surface PSCA levels in Pten CaP8 cells following YY1 knockdown and R1881 stimulation. The low levels of YY1 still present in the cells may be sufficient for interaction with AR and stimulation of PSCA expression. Alternately, AR may partially stimulate expression alone or in conjunction with other cofactors. Complete inhibition of PSCA expression in Pten CaP2 cells after YY1 knockdown suggests strong dependence on YY1 for R1881-mediated stimulation of PSCA expression. An earlier report showed that an AR-YY1 complex bound to the ARE in the human PSA promoter, and stimulated transcription [19]. Our *in vitro* data showed that promoter activity was abrogated when the mPSCA1 site adjacent to the ARE was mutated, suggesting that YY1 binding to this site mediates upregulation of PSCA together with AR. Our results do not exclude the possibility that an AR-YY1 complex is also binding to the ARE, and optimal stimulation of PSCA expression by AR may require both of these interactions.

Our results provide a potential explanation for the varied expression levels of PSCA in the different stages of prostate cancer. YY1 expression is elevated in PIN and intermediate stage disease [15], [18], and its interaction with AR may result in increased PSCA expression in PIN and well-differentiated cancers. Similarly, changes in YY1 levels together with changes in systemic androgen may modulate PSCA expression during tumor progression.

Increased reporter gene activity from the promoter construct with mutation of the distal YY1 binding site, mPSCA2, suggested that this is an inhibitory site. In an androgen-independent cell line, PC-3, knockdown of YY1 increased PSCA message. Although we cannot exclude the possibility that other mutations acquired by PC-3 cells may contribute to the downregulation of PSCA by YY1, our data strongly suggest that YY1 may inhibit PSCA expression in advanced prostate cancer. In murine and human androgen-dependent cell lines, YY1 knockdown did not increase endogenous PSCA message. However, by knocking down YY1 levels, we also prevented stimulation of PSCA transcription by AR. Thus, the potential increase in transcription by preventing YY1 binding to the inhibitory sites may be offset by the reduction in AR-mediated transcription.

The direct binding of YY1 to gene regulatory sequences can be either stimulatory or inhibitory, and depends on the associated recruited cofactors [31], [32]. Here we show that YY1 can both inhibit and stimulate the same promoter through interaction with different YY1 binding sites in the promoter, an observation also made previously for other promoters [35], [36]. Thus, regulation of PSCA by YY1 is likely to be context-dependent and modulated during tumor progression.

Although PSCA upregulation by androgen was defined some time ago, the mechanism by which PSCA expression is decreased in a subset of prostate cancers and in other epithelial cancers is unknown. Our data suggest repression of PSCA promoter activity by YY1 as one potential mechanism for reduced PSCA expression. The earlier description of regulation of the PSA promoter by an YY1-AR complex [19], together with the present work, suggests that YY1 may influence prostate cancer progression by regulation of at least two prostate cancer genes.

The role of YY1 in non-androgen dependent epithelial cancers such as bladder and pancreas, where PSCA is also expressed, is presently unknown. Elevated YY1 levels in these cancers may decrease PSCA levels by direct binding of YY1 to the inhibitory site in the promoter. Clinical data show that PSCA is up-regulated in bladder carcinoma *in situ* and down-regulated in invasive and advanced disease [37]. Recently, a single nucleotide polymorphism which reduced PSCA expression in human bladder [38] and gastric cancer [39] was associated with more aggressive disease. Down-regulation of PSCA expression by YY1 may similarly reduce PSCA expression and promote disease progression in these cancers. Further exploration of PSCA regulation by YY1 in other epithelial cancers will help to elucidate this mechanism.
